# ParaPep: a web resource for experimentally validated antiparasitic peptide sequences and their structures

**DOI:** 10.1093/database/bau051

**Published:** 2014-06-12

**Authors:** Divya Mehta, Priya Anand, Vineet Kumar, Anshika Joshi, Deepika Mathur, Sandeep Singh, Abhishek Tuknait, Kumardeep Chaudhary, Shailendra K. Gautam, Ankur Gautam, Grish C. Varshney, Gajendra P.S. Raghava

**Affiliations:** ^1^Cell biology and Immunology Division and ^2^Bioinformatics Centre, CSIR-Institute of Microbial Technology, Chandigarh-160036, India

## Abstract

ParaPep is a repository of antiparasitic peptides, which provides comprehensive information related to experimentally validated antiparasitic peptide sequences and their structures. The data were collected and compiled from published research papers, patents and from various databases. The current release of ParaPep holds 863 entries among which 519 are unique peptides. In addition to peptides having natural amino acids, ParaPep also consists of peptides having d-amino acids and chemically modified residues. In ParaPep, most of the peptides have been evaluated for growth inhibition of various species of *Plasmodium*, *Leishmania* and *Trypanosoma*. We have provided comprehensive information about these peptides that include peptide sequence, chemical modifications, stereochemistry, antiparasitic activity, origin, nature of peptide, assay types, type of parasite, mode of action and hemolytic activity. Structures of peptides consisting of natural, as well as modified amino acids have been determined using state-of-the-art software, PEPstr. To facilitate users, various user-friendly web tools, for data fetching, analysis and browsing, have been integrated. We hope that ParaPep will be advantageous in designing therapeutic peptides against parasitic diseases.

**Database URL:** http://crdd.osdd.net/raghava/parapep/

## Introduction

Parasites, including helminths and protozoa, are among the major contributors for parasitic diseases, which are a huge burden to mankind, particularly in tropical countries (1). Among the billions of people suffering from these diseases, more than a million die annually and one person in every four persons harbors parasitic worms. The currently used pharmaceutical treatments rely mainly on chemotherapeutic drugs. However, the increase in drug resistance (2, 3) and lack of effective vaccination makes the situation more complicated and alarming. Therefore, there is an urgent need to develop tools, as well as new drug candidates and strategies, to overcome the upcoming burden of parasitic diseases. One new approach is the use of therapeutic peptides to control the disease (4, 5). Peptide-based therapeutics have numerous advantages like high affinity, strong selectivity, low toxicity and high cell penetration (6, 7). In addition, with the advances in peptide synthesis, several chemical modifications can be made into peptides to optimize their physicochemical properties and affinity for a particular receptor (8).

Antiparasitic peptides (APPs) are small (∼5–30 amino acids) peptides, often derived from antimicrobial peptides (AMPs) (9). AMPs belong to a family of short peptides (<100 a.a.), which constitute a significant component of innate immunity (10). To date, several hundreds of AMPs have been discovered or synthesized and most of which are cationic and amphiphilic in nature. Natural AMPs can be obtained from both prokaryotes (e.g*.* bacteria) and eukaryotes (e.g. protozoa, fungi, plants, insects and animals). Being cationic in nature, AMPs show strong interaction with negatively charged components of lipidic membranes of microorganisms leading to the formation of ion channels and transmembrane pores (10). Their antimicrobial activity is mainly because of their tendency to disrupt membrane integrity, though few AMPs have internal targets as well (4). Earlier studies have suggested that many cationic AMPs are not toxic to normal eukaryotic cells and show a broad-spectrum activity against various parasites (4, 9). It is likely that the presence of anionic phospholipids at the outer leaflet of the membrane of parasites could be responsible for the relative specificity of APPs toward parasite over the host cells (4, 9, 11). Although the field of APPs is still in its infancy, the success of therapeutic peptides in other diseases like cancer has opened the door for APPs to reach clinics. In summary, APPs have emerged as promising therapeutic candidates against parasitic diseases, which have been reflected in a plethora of research articles showing the successful therapeutic application of APPs against various parasitic diseases (4). All this information is important but scattered in the literature, and thus is difficult to access.

To understand the properties and usefulness of APPs, there is a need to compile the information available in the literature pertaining to these peptides. To the best of authors’ knowledge, no database or resource provides comprehensive information about APPs. In this study, for the first time, an attempt has been made to collect and compile comprehensive information on APPs from literature and publicly available resources. We anticipate that this database will be useful for the scientific community working in the field of peptide therapeutics.

## Materials and methods

### Collection and compilation

We have collected peer-reviewed research articles published in past 30 years on APPs from various literature resources and search engines, including PubMed and Google Scholar. An extensive search was performed using various combinations of keywords like ‘anti-malarial peptides’, ‘anti-parasitic peptides’, ‘anti-plasmodial peptides’, using advanced search options. We obtained >350 research articles (Table 1), which were compiled to collect the required information like the type of peptides, type of assays, *in vitro*/*in vivo* model, activity of a peptide and its mode of action. In addition, APPs were also collected from patents and other databases like Collection of Antimicrobial Peptides (CAMP) (12), Antimicrobial Peptide Database (APD2) (13) and Dragon Antimicrobial Peptide Database (DAMPD) (14). Multiple entries of a single peptide have been made if the peptide has been tested for growth inhibition of more than one parasite or the peptide has been tested at different concentrations. Finally, information is compiled in 863 entries where each entry contains hyperlinks for more information on these peptide entries. The database provides extensive cross-references and web interface for data retrieval.

**Table 1.** List of keyword combinations used for the data collection from different sources

Keyword in title/abstract	Number of articles
PubMed (11 July 2013)	
Antimalarial peptide	138
Antiparasitic peptide	57
Antiplasmodial peptide	17
Antiparasite peptides	12
Antitrypanosomal peptide	8
Antileishmanial peptide	11
Anti leishmania peptide	8
Leishmanial peptide	24
Anti-babesial peptide	1
Antischistosomal peptide	4
Anti lyme disease peptide	0
Anti hookworm peptide	0
Sleeping Sickness peptide	25
Anti toxoplasmal peptide	1
Ascariasis peptide	2
Giardiasis peptide	7
Scabies peptide	6
Trypanosoma peptides	29
Google scholar (11 July 2013)	
Antimalarial peptide	19
Antiplasmodial peptide	5
Antiparasitic peptide	41

### Database architecture and web interface

**Figure 1.**
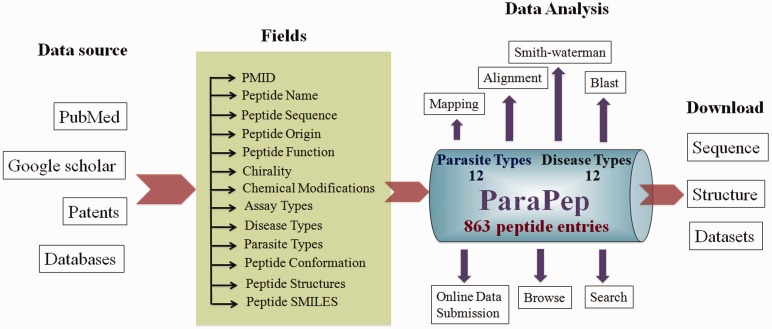
Architecture of ParaPep database.

We have built ParaPep database using the standard platform based on Linux-Apache-MySQL-PHP (LAMP). In this database, we used Red Hat Linux (version 6.2) as the operating system, Apache (version 2.2.17) as HTTP Server and MySQL (version 14.12) for managing data. The front-end was developed using HTML (version 5), PHP (version 5.2.14) and Javascript (version 1.7), and MySQL supported the back-end. All common gateway interface and database interfacing scripts were written in the PHP and PERL (version 5.10.1) programming languages. The architecture of ParaPep database is shown in Figure 1.

### Organization of data

The information maintained in ParaPep database can be divided broadly in two main categories: (i) primary information and (ii) secondary information. Primary data have been extracted manually from literature, which include APP sequences and other related experimental information like assay types, parasite type and mode of action. Following is a brief description of information stored in the ParaPep database.

#### Primary data

Each peptide is assigned a unique entry number, and detailed information about each peptide has been provided. Each entry contains the following major fields: (i) name of peptide, (ii) amino acid sequence of peptide, (iii) chirality/topology of peptide (L/D and linear/cyclic), (iv) details of chemical modification (e.g. ornithine, β-alanine), (v) nature (e.g. antimicrobial) of peptide, (vi) origin of peptide (e.g. Snake venom), (vii) antiparasitic activity (e.g. IC_50_ = 75 µM), (viii) modifications at *N*- and *C*-terminus of peptide (e.g. acetylation/amidation), (ix) type of assay used to determine the activity (e.g. [^3^H]Hypoxanthine incorporation assay), (x) mode of action (e.g. permeabilization of the cell membrane), (xi) type of model system used for assay (e.g. *in vitro*/*in vivo*), (xii) type of parasite (e.g. *P. falciparum*) and (xiii) type of disease (e.g. Malaria).

#### Secondary information/data

Secondary information was derived from primary data. We derived different types of information, including structural annotation of each peptide. Structure of a peptide plays a vital role in its function, and thus, we made a systematic attempt to derive the structure of each peptide from its sequence. We used the following steps to generate the structure of all peptides including modified ones. First, similarity search was performed for each peptide in ParaPep against Protein Data Bank (PDB), and the structure was assigned if identical peptide is already available in PDB. Second, for all peptides whose identical peptides are not available in PDB, we used state-of-the-art algorithm PEPstr (15) for predicting the tertiary structure of peptides from amino acid sequence. We were able to predict the structure of all peptides containing only natural amino acids.

Many APPs in ParaPep consist of nonnatural, as well as chemically modified, residues, which cannot be predicted using PEPstr. To the best of authors’ knowledge, no method is available online that can predict the tertiary structure of peptides having nonnatural or modified amino acids. As the structure may play an important role in the function of APPs, it becomes imperative to have structural insights of these peptides. In this study, we extended the use of PEPstr for predicting the tertiary structure of peptides with nonnatural amino acids. Given a query sequence, PEPstr extracts the secondary structure information using PSIPRED (16) and beta turn types information using BetaTurns (17) and integrates both types of information to obtain an initial structure, which is followed by energy minimization and molecular dynamics using AMBER 11 (18).

We have predicted tertiary structures of peptides having natural amino acids, d-amino acids and end modifications like acetylation/amidation, as well as peptides having ornithine as modified amino acid, by extending the use of AMBER 11 (18) in PEPstr algorithm. For changing the stereochemistry of a residue in D-form, the flip command of AMBER 11 was used. For nonnatural residues like ornithine, force field library for that residue was used in AMBER 11 (19). In our database, we maintain the tertiary structure of peptides in PDB format. In addition, we have predicted the structure of peptides with diverse chemical modifications. For any peptide with chemical modification, first its backbone structure was predicted using PEPstr followed by the manual incorporation of modified moiety using ChemDraw chemical drawing software, and finally energy minimization and molecular dynamics of the structure were done with default parameters using MM2 software inbuilt in ChemDraw 3D software.

To assign the secondary structural states of the peptides, we used the DSSP software (20), which is a well-established method and assigns eight secondary structural states using PDB file as input. We have also maintained structure of peptides in Simplified Molecular-Input Line-Entry System (SMILES) notation. SMILES of ∼100 peptides were compiled from the literature. We generated SMILES for the rest of the peptide sequences from predicted structure using Open Babel software (21), which generates SMILES notation using tertiary structure in PDB format as input. During data curation, we noticed that in many cases, peptide sequences were not available in the research articles, but the structure of peptides were represented in the form of images. We used online service of Optical Structure Recognition Application (22) to get SMILES notation of these structures. Once SMILES were generated, we converted them into PDB format using Open Babel software. Finally, energy minimization and molecular dynamics were carried out using MM2 software. The minimization software could not minimize structures of few peptides, and therefore, the structures of these peptides are not available.

### Integration of web tools

A variety of user-friendly tools have been integrated in ParaPep, which facilitates users in accessing desired information from the database. Numerous tools have been integrated to facilitate various types of data analyses. Following are the major tools provided with the ParaPep:

#### Data retrieval or search tools

This module of ParaPep has the following four search options: Simple, Complex, Peptide and SMILES. In case of Simple search, the server allows users to perform a search on any field of the database like peptide name, peptide sequence, parasite type, disease type and antiparasitic activity. This option allows displaying any or all fields of entries in a resultant output of query search. Complex search is designed to perform advanced or conditional search in ParaPep using simultaneous multiple queries with Boolean expressions (e.g. AND & OR). Peptide search option has been developed to search identical peptide sequences in the database. In ParaPep, one can search structure of a peptide using SMILES search option. It allows various options like superstructure, substructure and peptide search.

#### Browsing

To access the data in ParaPep, various browsable tables that are linked from the database homepage have been provided. User can browse on five major fields: (i) chirality of peptides, (ii) nature of peptides, (iii) length of peptides, (iv) disease type and (v) parasite type. In ParaPep, we have compiled peptides tested for the growth inhibition of 12 different types of parasites each causing a specific disease. User can browse data for a particular parasite or disease. For instance, user can browse all the peptides tested for the growth inhibition of *P. falciparum* causing Malaria. In addition, we have also compiled information regarding various types of *in vitro* and *in vivo* assays used to evaluate the activity of APPs. Browsing facility based on assay types facilitates users to fetch APPs tested for a particular assay (e.g. [^3^H]Hypoxanthine incorporation assay). We have also compiled the nature of APPs and developed a browsing interface, which is helpful to browse APPs having specific nature like antimicrobial peptides. Because the users may be interested to know the length and chirality of APPs, browsing interfaces to extract data based on these two features have therefore been developed.

### Sequence alignment based web tools

To understand the relation between sequence similarity functions, a number of tools based on alignment have been integrated. This includes similarity search tools, where the user can search similar sequence in ParaPep for their query sequence, using BLAST and Smith-Waterman algorithm. Following is a brief description of tools integrated in this module.

***BLAST search***: To perform similarity search against the database, we integrated popular software BLAST, which is commonly used for similarity search. Users can submit their peptide in FASTA format with desired or default parameters of BLAST (18). The server performs BLAST search for user’s query sequence against peptide sequences in the database.

***Smith–Waterman algorithm***: Smith–Waterman algorithm (23) has been integrated because it performs similarity search more effectively in case of small peptides. Users can search multiple peptide sequences at a time by submitting sequences in FASTA format.

***Alignment***: It facilitates users in generating alignment between users’ query sequences or peptide sequences in ParaPep. The user can get aligned sequences by submitting multiple FASTA sequences in the sequence box and peptide IDs of ParaPep database in the ID box. The user also has the option to upload a PDB file and align its structure with the structure of the peptide whose ID is provided in the box.

***Mapping***: This tool assists the users to map APPs on their query peptide. There are two options for mapping: (i) sub-search and (ii) super-search. Sub-search allows the users to map query peptide against all APPs of ParaPep, whereas super-search allows mapping of protein sequence against ParaPep and identify segments that are identical to APPs.

## Results

**Figure 2.**
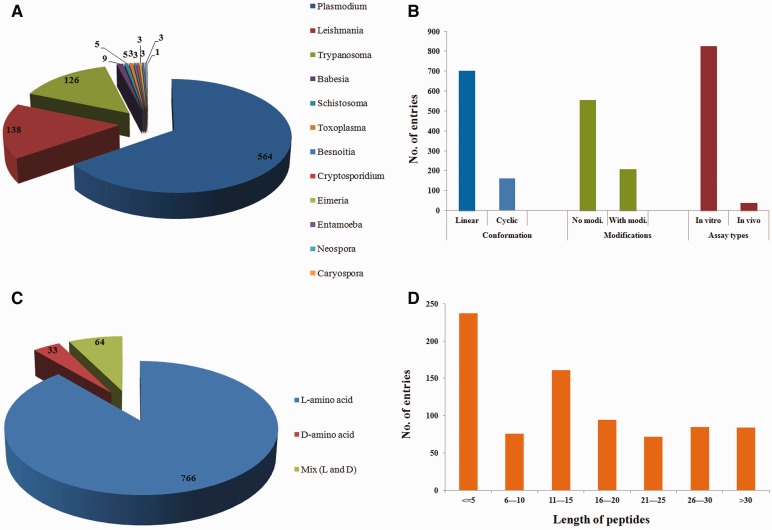
Distribution of peptides based on (**A**) parasite types, (**B**) topology, chemical modifications and assays types, (**C**) stereochemistry and (**D**) length.

The ParaPep database consists of 863 entries (Figure 1) of experimentally validated APPs. It covers data of growth inhibition studies on 12 parasite types each causing a specific disease (Figure 2A). Most of the entries (564 entries) have been made for peptides tested against *Plasmodium* species causing Malaria followed by *Leishmania* (138 entries) and *Trypanosoma* (126 entries, Figure 2A). During data collection and compilation, we have noticed that many peptides have been evaluated against more than one parasite (e.g*.* *Plasmodium* and *Leishmania*). Therefore, we have made multiple entries of a single peptide if it has been tested against more than one parasite. Thus, ParaPep has 519 unique peptide sequences, which are stored in 863 entries. Most of the peptides have been tested *in vitro*, whereas 38 peptides have been evaluated *in vivo* (Figure 2B). In the literature, different types of *in vitro* assays have been reported to determine the antiparasitic activity of these peptides. For instance, to determine the anti-plasmodium activity, four types of assays have been reported that includes SYBR Green-based assay, [^3^H]Hypoxanthine incorporation assay, lactate dehydrogenase release assay and Giemsa staining assay. Pertaining to this aspect, ∼149 entries have been made for [^3^H]Hypoxanthine incorporation assay followed by Giemsa staining (146 entries) and MTT (3-[4,5-dimethylthiazol-2-yl]-2,5 diphenyl tetrazolium bromide) assay (124 entries).

Because the stability of peptides is a major concern, especially when tested *in vivo*, most of the studies have reported the use of peptides having nonnatural amino acids or the peptides with various chemical modifications. We have also stored this information and compiled peptides based on their topology (linear/cyclic) and chirality, i.e. L/D/Mix (both L and D). ParaPep contains 703 entries of linear peptides, while 160 entries have information about cyclic peptides (Figure 2B). In total, 208 peptide entries have been included, which provide information about APPs having chemical modifications (Figure 2B). We have compiled 766 entries of peptides containing only L-amino acids, 33 entries of peptides consist of only D-amino acids and 64 entries of peptides have both L- and D-amino acids (Figure 2C). We have also included information related to the length of the peptides. In ParaPep, 237 peptides have length less than 5, whereas 161 peptides have length between 11 and 15 (Figure 2D). Peptides in ParaPep database belong to diverse classes of therapeutic peptides and have different functions that include antimicrobial, antiparasitic, anticancer, cell penetrating, antiviral peptides, etc. However, most of the entries in ParaPep belong to AMPs (390 entries) followed by antimalarial peptides (103). In most of the studies, the modes of action of APPs have also been reported that include destabilization of the plasma membrane of parasite and inhibition of internal targets. Because this information could be useful for users, in the current release of ParaPep, we have also incorporated information about the mode of action of various APPs.

## Discussion

Parasitic diseases like malaria have become serious threats to the life of billions of human beings worldwide. Chemotherapeutic drugs, which have been the principle mode of treatment for the past 30 years, are now becoming ineffective owing to the emergence of drug resistance in these bugs. Accordingly, the attention of the scientific community is now shifted to finding new alternative means, which are more effective and specific in therapy (4). Recently, peptides are emerging as attractive candidates for the development of therapeutic agents against parasitic diseases (4). This growing interest in therapeutic peptide-based research is primarily owing to: (i) recent advancements in peptide synthesis and (ii) advantages of peptides over small molecules, like high selectivity, low toxicity and high tissue penetration (7). In addition, ease of modifications and overall low production cost makes peptides more popular compared with antibodies and therapeutic proteins.

Over the past decade, a plethora of studies has reported the antiparasitic properties of many therapeutic peptides (4, 5, 9, 11). The results of these studies were promising and raised a hope for the fight against these devastating diseases. Keeping in mind the huge pharmacological importance of therapeutic peptides, many databases of therapeutic peptides have been developed so far that include CPPsite (24), APD2 (13), CAMP (12), Brainpeps (25), Quorumpeps (26), TumorHope (27), Hemolytik (28) and DAMPD (14). However, no attempt has been made to catalog APPs and to understand the properties of these peptides. Therefore, in this study, we have made a maiden attempt to develop a repository, which provides comprehensive information related to APPs. Besides, ParaPep will also be useful to develop various prediction methods for designing and predicting better APPs. The structural information and SMILES of APPs can be exploited to perform various types of analyses like quantitative structure activity relationships.

### Summary and future perspectives

In summary, ParaPep is a much-needed resource of experimentally validated APPs. This is the first version of the database that contains comprehensive information related to APPs. We have made an attempt to provide the structures of APPs. The database provides a user-friendly interface with various tools, which facilitates the data analysis and browsing. In addition, ParaPep is interlinked with other resources in the field. ParaPep is designed with room for the users to submit new entries of APPs online by filling HTML form. To maintain a high level of quality, our team will confirm the validity of each new entry before incorporating into ParaPep. In addition, ParaPep team will continuously collect the novel APPs from the research articles and patents to keep ParaPep up-to-date. We believe that ParaPep will be a useful resource for both experimental and bioinformatics researchers working in the field of therapeutic peptides designing. Our team will update the database on regular intervals, as soon as the data regarding the new antiparasitic peptides will be available.
